# Global Trends in ADHD Medication Use: Multiple Contexts and Rising Concerns—A Narrative Review

**DOI:** 10.3390/jcm14207338

**Published:** 2025-10-17

**Authors:** Marcin Rzeszutek, Tomasz Wolańczyk

**Affiliations:** Department of Child Psychiatry, Medical University of Warsaw, Żwirki i Wigury 61, 02-091 Warsaw, Poland; psychiatria.dsk@uckwum.pl

**Keywords:** methylphenidate, amphetamine, atomoxetine, guanfacine, dexamfetamine, lisdexamfetamine, utilization, consumption, prevalence, stimulants

## Abstract

Attention-deficit/hyperactivity disorder (ADHD) is a neurodevelopmental condition frequently treated with pharmacological interventions, most commonly stimulants such as methylphenidate and amphetamines, alongside non-stimulant options. This narrative review, based on 31 publications and five national drug utilization registers, summarizes global trends in ADHD medication use since 2000. Across most countries, prevalence of ADHD medication use increased steadily, with the sole exception of the Netherlands, where recent declines were observed. The highest prevalence of ADHD medication use was consistently found among older children and adolescents. While boys showed higher values of prevalence of ADHD medication use than girls in childhood, faster increases among females resulted in reversed gender ratios in several adult populations. Methylphenidate remained the most widely prescribed drug, although the use of lisdexamfetamine and guanfacine has expanded in recent years. Variations in national guidelines, diagnostic frameworks, healthcare access, and sociocultural acceptance of pharmacotherapy contributed to observed differences across regions. Increasing use of ADHD medications raises important questions about equitable access to treatment, potential overdiagnosis, and the risk of stimulant misuse. These findings highlight the need for continued monitoring of utilization patterns to ensure safe, rational, and equitable ADHD care worldwide.

## 1. Introduction

Attention-Deficit Hyperactivity Disorder (ADHD) medications are a group of substances used as one of the treatment methods for ADHD symptoms such as inattention, impulsivity, and hyperactivity. These medications can be divided into two subgroups: stimulants and non-stimulants. Psychostimulants include methylphenidate (MPH), amphetamine (AMP), and its derivatives—dexamfetamine (DEX) and lisdexamfetamine (LDX). Non-stimulant medication includes atomoxetine (ATX), clonidine (CLO), and guanfacine (GUA).

Guidelines from European countries, North America, and Australia recommend incorporating pharmacological interventions as part of a multimodal treatment program alongside non-pharmacological interventions [1,2,3,4].

The global prevalence of ADHD was estimated at 3.4% among children and adolescents [5], while among adults, 2.6% were individuals with childhood-onset ADHD, and 6.8% were adults who met ADHD criteria regardless of whether onset occurred in childhood [6].

ADHD is a neurodevelopmental disorder whose symptoms may persist throughout life with varying intensity. Estimates of symptom persistence into adulthood vary widely, ranging from 5% to 75% [7].

To establish a diagnosis of ADHD, the symptoms presented by the patient should meet the diagnostic criteria of the DSM-5 or ICD-11, significantly impair functioning, and occur in at least two important settings. The diagnostic process should be based on a comprehensive clinical and psychosocial evaluation, and should also take into account the patient’s developmental and psychiatric history, observer reports, and assessment of the person’s mental state [1].

Pharmacoepidemiological studies provide a better understanding of medication use patterns and their alignment with clinical guidelines, enable the identification of overuse or underuse of medications, and facilitate health policy planning and organization, as well as the promotion of rational drug use. They reveal differences in medication consumption between females and males, patients from different age groups, and those living in various regions of a country. This allows for the identification of underserved social groups and supports efforts to ensure equitable access to treatment. Moreover, analyzing drug use trends helps assess the impact of external factors on medication use.

The two most fundamental pharmacoepidemiological measures are incidence and prevalence [8]. However, other common measures used to illustrate drug use trends include the number of prescriptions or defined daily dose (DDD) per 1000 inhabitants. The diversity of measures and data sources allows researchers to capture various aspects of drug consumption and identify factors influencing observed trends over time. However, it can also introduce bias and complicate comparisons of trends across different regions and population groups.

The aim of this narrative review is to summarize global trends in ADHD medication use and to explore how these trends vary across age groups and between males and females.

## 2. Materials and Methods

A search of the Medline database was conducted using the following keywords: trend, use, stimulants, methylphenidate, ADHD medication, utilization, and consumption. The complete search strings used for all databases are provided in the Supplementary Materials (Supplement S1) to enhance transparency and reproducibility of the review. Additionally, the reference lists of the publications included in the review were screened. The selection was guided by the following inclusion criteria:

1. Articles published in English.
2. Studies based on nationwide patient registries were prioritized. When national-level data were not available, studies using regional datasets or research samples extrapolated to the national population were included.
3. Availability of full-text versions.
4. Publications reporting medication use at a minimum of two time points.
5. To allow for comparison of trends between countries, as well as between males and females and across age groups, only studies that reported medication use in terms of prevalence were included. Prevalence was defined as the number of individuals who were prescribed or self-reported taking ADHD medication at least once during the year, expressed relative to the total population.
6. Studies with observation periods including years after 2000.
7. Publications analyzing solely preschool-aged populations were excluded.
8. Studies describing trends in medication use for only one gender were excluded.

As part of the methodological approach, a literature search was conducted in MEDLINE to identify relevant publications, and for each country represented, it was verified whether national drug utilization data were publicly and freely available. When such data were accessible, national-level consumption was described based on these official registers.

Prevalence values from individual studies were converted to rates per 1000 individuals.

To help readers assess the quality and reliability of the reported country-level trends, we applied a descriptive grading system based on data completeness, population coverage, and validation status. Each data source was classified as high (national prescription registry), moderate (administrative claims or large-scale primary care database), or low (survey-based data). This grading is presented in Supplementary Table S1.

The review was conducted in July 2025.

One additional publication identified during the revision process was subsequently included in the review.

## 3. Results

32 publications and 5 national drug utilization registers were included in this review. Their characteristics are summarized in Table 1. Across all countries represented in the included studies and registers, the prevalence of ADHD medication use increased over the available observation periods, with the sole exception of the Netherlands, where a decline was observed in recent years.

The Nordic countries—Sweden, Norway, Denmark, and Iceland—provide open access to national drug utilization registers covering nearly 100% of their populations [9], Australia also offers free and direct public access to prescription data through government reports.

**Table 1:** Summary of included studies and registers.

Studies					
Study	Years Covered	Countries Included	Data Source	Age	Drugs
Lopez-Leon, Sandra et al. Psychotropic medication in children and adolescents in the United States in the year 2004 vs. 2014 [10].	2004 and 2014	US	MarketScan Commercial Claims and Medicare database and the MarketScan Medicaid database	2–18	MPH, DEX, ATX, CLO, GUA
Zuvekas, S.H., Vitiello, B. Stimulant medication use in children: a 12-year perspective [11].	1996–2008	US	Medical Expenditure Panel Survey (MEPS)	0–18	Stimulants (MPH, DEX-MPH, AMP, DEX)
Zuvekas, S.H., Vitiello, B., Norquist, G.S. Recent trends in stimulant medication use among U.S. children [12].	1997–2002	US	Medical Expenditure Panel Survey (MEPS)	0–18	Stimulants (MPH, DEX-MPH, AMP, DEX)
Moore, T.J., Wirtz, P.W., Kruszewski, S.P., Alexander, G.C. Changes in medical use of central nervous system stimulants among US adults, 2013 and 2018: a cross-sectional study [13].	2013 and 2018	US	Medical Expenditure Panel Survey (MEPS)	>18	Stimulants (MPH, DEX-MPH, AMP, DEX, LDX)
Cox, E.R., Halloran, D.R., Homan, S.M., Welliver, S., Mager, D.E. Trends in the prevalence of chronic medication use in children: 2002–2005 [14].	2002 and 2005	US	Express Scripts, Inc.	5–19	ADHD medication
Danielson, M.L., Bohm, M.K., Newsome, K. et al. Trends in Stimulant Prescription Fills Among Commercially Insured Children and Adults—United States, 2016–2021. MMWR Morb Mortal Wkly Rep 2023;72:327–332 [15].	2016–2021	US	Merative MarketScan Commercial Database	5–64	MPH, AMP
Raman, S.R., Man, K.K.C., Bahmanyar, S., Berard, A., Bilder, S., Boukhris, T. et al. Trends in attention-deficit hyperactivity disorder medication use: a retrospective observational study using population-based databases [16].	2001–2015	Japan, Taiwan, Australia, Canada, US (2 sites), Finland, France, United Kingdom, Spain	Data included electronic patient-level records of prescriptions and dispensations from clinical and insurance claim systems.	>2, but 3–11 for Canada	MPH, DEX, LDX, ATX, GUA, CLO
Bachmann, C.J., Wijlaars, L.P., Kalverdijk, L.J., Burcu, M., Glaeske, G., Schuiling-Veninga, C.C.M., Hoffmann, F., Aagaard, L., Zito, J.M. Trends in ADHD medication use in children and adolescents in five western countries, 2005–2012 [17].	2005–2012	UK, Germany, Netherlands	Germany: BARMER GEK, Netherlands: IADB.nl, UK: The Health Improvement Network (THIN).	0–19	MPH, AMP, ATX
Hartz, I., Madsstuen, N.H.H., Andersen, P.N., Handal, M., Odsbu, I. Nationwide trends in the use of ADHD medications in the period 2006–2022: a study from the Norwegian prescription database [18].	2006–2022	Norway	Norwegian Prescription Database (NorPD)	6–64	MPH, DEX, LDX, ATX, GUA
Furu, K., Karlstad, Ø., Zoega. H., Martikainen, J.E., Bahmanyar, S., Kieler, H., Pottegård, A. Utilization of Stimulants and Atomoxetine for Attention-Deficit/Hyperactivity Disorder among 5.4 Million Children Using Population-Based Longitudinal Data [19].	2008–2012	Norway, Denmark, Sweden, Finland, Iceland,	Norwegian Prescription Database, Danish National Prescription Registry, Swedish Prescribed Drug Register, Finnish Prescription Register, Icelandic Medicines Registry	0–17	MPH, DEX, LDX, ATX, GUA
Karlstad, Ø., Zoëga, H., Furu, K., Bahmanyar, S., Martikainen, J.E., Kieler, H., Pottegård, A. Use of drugs for ADHD among adults-a multinational study among 15.8 million adults in the Nordic countries [20].	2008–2012	Norway, Denmark, Sweden, Finland, Iceland,	Norwegian Prescription Database, Danish National Prescription Registry, Swedish Prescribed Drug Register, Finnish Prescription Register, Icelandic Medicines Registry	18–64	MPH, DEX, AMP, ATX
Sørensen, A.M.S., Wesselhöeft, R., Andersen, J.H., Reutfors, J., Cesta, C.E., Furu, K., Hartz, I., Rasmussen, L. Trends in use of attention deficit hyperactivity disorder medication among children and adolescents in Scandinavia in 2010–2020 [21].	2010–2020	Norway, Denmark, Sweden	Norwegian Prescription Database, Danish National Prescription Registry, Swedish Prescribed Drug Register	5–19	MPH, DEX, LDX, ATX, GUA
Hartz, I., Skurtveit, S., Steffenak, A.K., Karlstad, O., Handal, M. Psychotropic drug use among 0–17 year olds during 2004–2014: a nationwide prescription database study [22].	2004–2014	Norway	Norwegian Prescription Database	<18	Stimulants
Lillemoen, P.K., Kjosavik, S.R., Hunskår, S., Ruths, S. Prescriptions for ADHD medication, 2004–2008 [23].	2004–2008	Norway	Norwegian Prescription Database	All ages	MPH, AMP, DEX, ATX
Hodgkins, P., Sasané, R., Meijer, W.M. Pharmacologic treatment of attention-deficit/hyperactivity disorder in children: incidence, prevalence, and treatment patterns in the Netherlands [24].	2000–2007	Netherlands	PHARMO Record Linkage System (RLS)	6–17	MPH, DEX, ATX
Ringeling, L.T., Srivastava, A., Gangapersad, R.N. et al. ADHD medication dispensing trends in Dutch youth before and after the implementation of the Youth Act [25].	2010–2022	Netherlands	InterAction Database (IADB)	0–19	Stimulants and GUA
Trip, A.M., Visser, S.T., Kalverdijk, L.J., de Jong-van den Berg, L.T. Large increase of the use of psycho-stimulants among youth in the Netherlands between 1996 and 2006 [26].	1996–2006	Netherlands	IADB	0–19	Stimulants
Kraut, A.A., Langner, I., Lindemann, C., Banaschewski, T., Petermann, U., Petermann, F., Mikolajczyk, R.T., Garbe, E. Comorbidities in ADHD children treated with methylphenidate: a database study [27].	2004–2006	Germany	The German Pharmacoepidemiological Research Database (GePaRD)	3–17	MPH
Abbas, S., Ihle, P., Adler, J.B., Engel, S., Günster, C., Linder, R., Lehmkuhl, G., Schübert, I. Psychopharmacological Prescriptions in Children and Adolescents in Germany [28].	2004–2012	Germany	Data from two health insurance companies	0–17	Psychostimulants (ATX included)
Ehrhardt, C., Boucherie, Q., Pauly, V., Braunstein, D., Ronflé, E., Thirion, X., Frauger, E., Micallef, J. Methylphenidate: Gender trends in adult and pediatric populations over a 7 year period [29].	2005–2011	France	French general health insurance system (FGHIS)	All ages	MPH
Fond, G., Pauly, V., Brousse, Y. et al. Mental Health Care Utilization and Prescription Rates Among Children, Adolescents, and Young Adults in France [30].	2016–2023	France	French National Health Insurance Database (SNDS)	0–25	MPH
Stuhec, M., Locatelli, I. Attention deficit hyperactivity disorder pharmacotherapy in Slovenian adults: a population-based study [31].	2003–2015	Slovenia	NHI—Health Insurance Institute of Slovenia	>18	MPH, ATX
Stuhec, M., Locatelli, I. Age-related pharmacotherapy of attention deficit hyperactivity disorder in Slovenia in children and adolescents: A population-based study [32].	2003–2015	Slovenia	NHI—Health Insurance Institute of Slovenia	2–18	MPH, ATX
Boland, F., Galvin, R., Reulbach, U., Motterlini, N., Kelly, D., Bennett, K., Fahey, T. Psychostimulant prescribing trends in a paediatric population in Ireland: a national cohort study [33].	2002–2011	Ireland	GMS (General Medical Services) pharmacy claims database	0–15	MPH, DEX, LDX, Modafinil
Beau-Lejdstrom, R., Douglas, I., Evans, S.J., Smeeth, L. Latest trends in ADHD drug prescribing patterns in children in the UK: prevalence, incidence and persistence [34].	1992–2013	UK	CPRD (Clinical Practice Research Datalink)	0–15	MPH, DEX, ATX, Modafinil
McCarthy, S., Wilton, L., Murray, M.L., Hodgkins, P., Asherson, P., Wong, I.C. The epidemiology of pharmacologically treated attention deficit hyperactivity disorder (ADHD) in children, adolescents and adults in UK primary care [35].	2003–2008	UK	The Health Improvement Network (THIN)	>6	MPH, DEX, ATX
McKechnie, D.G.J., O’Nions, E., Dunsmuir, S., Petersen, I. Attention-deficit hyperactivity disorder diagnoses and prescriptions in UK primary care, 2000–2018: population-based cohort study [36].	2000–2018	UK	IQVIA Medical Research Data	3–99	MPH, AMP + DEX, DEX, LDX, ATX, GUA
Butt, D.A., Stephenson, E., Kalia, S., Moineddin, R., Tu, K. Patient visits and prescriptions for attention-deficit/hyperactivity disorder from 2017–2021: Impacts of COVID-19 pandemic in primary care [37].	2017–2021	Canada	UTOPIAN Data Safe Haven	5–55	MPH, LDX, AMP, DEX, ATX, GUA, Modafinil
Morkem, R., Patten, S., Queenan, J., Barber, D. Recent Trends in the Prescribing of ADHD Medications in Canadian Primary Care [38].	2005–2015	Canada	Canadian Primary Care Sentinel Surveillance Network (CPCSSN)	0–65	MPH, AMP, LDX, DEX, ATX, Modafinil
Brett, J., Karanges, E.A., Daniels, B. et al. Psychotropic medication use in Australia, 2007 to 2015: Changes in annual incidence, prevalence and treatment exposure [39].	2006–2015	Australia	Pharmaceutical Benefits Scheme (PBS)	>17	MPH, DEX, ATX
Song, I., Lee, M.S., Lee, E.-K., Shin, J.-Y. Patient and provider characteristics related with prescribing of ADHD medication: Nationwide health insurance claims database study in Korea [40].	2007–2011	South Korea	Korea Health Insurance Review and Assessment Service (HIRA)	0–17	MPH, ATX
Hoshen, M.B., Benis, A., Keyes, K.M., Zoëga, H. Stimulant use for ADHD and relative age in class among children in Israel [41].	2006–2011	Israel	Clalit Health Services dataset	6–17	MPH, ATX, DEX, AMP
Registers					
The Swedish Prescribed Drug Registry [42].	2006–2024	Sweden	-	0–79	AMP, DEX, LDX MPH, ATX, GUA
Norhealth (www.norgeshelsa.no, accessed on 14 July 2025) [43], The Norwegian Prescription Database [44]	2004–2021	Norway	-	0–79	DEX, LDX, MPH, ATX, GUA
The Danish Health Data Authority, medstat.dk and date [45].	1999–2024	Denmark	-	0–79	DEX, LDX, MPH, ATX, GUA
The Icelandic Pharmaceutical Database [46]	2005–2024	Iceland	-	0–79	AMP, DEX, LDX, MPH, ATX, GUA
The Australian Institute of Health and Welfare (AIHW) (2025) ADHD medications dispensed 2004–2005 to 2023–2024 [47]	2004–2024	Australia	Pharmaceutical Benefits Scheme (PBS)	All	MPH, DEX, LDX, ATX, GUA

### 3.1. North America

#### 3.1.1. United States (US)

Between 1996 and 2008, stimulant use was most prevalent among children aged 6–12. It declined slightly among those aged 0–5, while increasing significantly among adolescents aged 13–18 [11,12]. Another study reported a peak around ages 10–11 in 2008 and 2014 [10]. In a study analyzing prescription stimulant trends between 2016 and 2021, it was reported that the highest percentage of male enrollees with one or more prescription stimulant fills was observed in the 10–14 age group, whereas for females the highest percentages were in the 20–24 and 15–19 age groups [15].

Among children and adolescents, the average annual percentage change (AAPC) between 2016 and 2020 in the age groups 5–9, 10–14, and 15–19 was negative for both sexes, except for girls aged 10–14 and 15–19, for whom the values were 0 and 0.1, respectively. In contrast, the annual percentage change (APC) from 2020 to 2021 for girls was positive across all age groups, except for the 5–9 age group, where it remained 0. For boys, the APC from 2020 to 2021 was negative across all age groups [15].

Among adults, the AAPC between 2016 and 2020 was positive across all age groups, except for females and males aged 20–24, where it was negative, and males aged 60–64, where it was 0. The APC from 2020 to 2021 among adults was positive across all age groups, except for males aged 60–64, where it remained 0 [15].

Overall, in the US, males are more likely to receive ADHD medication prescriptions than females [10,12,14,15]. Although in 2005, the prevalence of ADHD medication use remains higher among males, a faster increase was observed among females, particularly in the 15–19 age group [14]. In 2018, a reversal in gender predominance among adults has been observed, with females surpassing males [13,15].

MPH remains the most commonly prescribed stimulant among children [10]. However, a decline in the use of ATX and DEX, and an increased use of other medications such as LDX, dexmethylphenidate (DEX-MPH), CLO, and GUA, has been reported [10]. In adults, amphetamine products are more widely used compared to methylphenidate products, with slower growth observed in MPH use [13].

Comparative data from large-scale databases, such as Medicaid and MarketScan, demonstrate disparities in stimulant prescribing patterns. MPH is most commonly used in the Medicaid population, while amphetamines dominate among privately insured groups. What’s more, a Medicaid population showed higher estimates than those who were privately insured through employers (MarketScan) [16].

#### 3.1.2. Canada

In trend analysis covering 2005 to 2015, prescribing rates increased most notably among young adults aged 18–25. In adults aged 18–65, prevalence rose from 3.2 per 1000 in 2005 to 12.9 per 1000 in 2015, reflecting a 4-fold increase [38].

The highest prevalence was recorded in the 15–19 age group in 2017 and 2019, and in the 20–24 age group in 2018, 2020, and 2021. Although males consistently had higher prevalence rates than females, the gender gap narrowed over time. In 2017, prevalence was 17.3 per 1000 in males and 9.3 per 1000 in females. In 2021, these values rose to 25.6 per 1000 and 18.8 per 1000, respectively [37].

MPH remained the most prescribed substance overall, though its market share slightly declined from 64.5% in 2005 to 60.9% in 2015, while amphetamine-based medication and ATX gained popularity. Among children aged 6–17, the male/female ratio (M/F ratio) dropped from 3.29 in 2005 to 2.7 in 2015 [38].

### 3.2. Northern Europe

#### 3.2.1. Scandinavian Countries

Three studies reporting on trends in ADHD medication use across Scandinavian countries were included in the review. Two of these covered all five Nordic countries (Denmark, Finland, Iceland, Norway, and Sweden), while one focused specifically on Sweden, Norway, and Denmark.

Among children and adolescents (0–17) across the five Nordic countries, the prevalence of ADHD medication use increased from 8.9 per 1000 in 2008 (48,296 of 5.42 million) to 14.4 per 1000 in 2012 (77,604 of 5.40 million) [19]. In 2008, the highest prevalence was observed among boys aged 6–12 years (19.9 per 1000), while in 2012 the highest shifted to boys aged 13–17 (32.7 per 1000). For girls, the highest prevalence in both years was consistently seen in the 13–17 age group, with peak age at 16, while for boys it was at 12. Across the period, the increase was more pronounced among girls [19]. MPH was the dominant substance used [19].

In adults (aged 18–64 years), the prevalence of ADHD medication use increased between 2008 and 2012—from 2.4 to 5.3 per 1000 in males and from 1.8 to 4.4 per 1000 in females. The highest rates in 2012 were observed among 18-year-olds (17.2 per 1000 in males and 12.8 in females), with progressively lower prevalence seen in older adult age groups. The M/F ratio declined from 1.33 in 2008 to 1.20 in 2012. MPH remained the most frequently used drug, with an increasing proportion of extended-release (ER) formulations and a decline in immediate-release (IR) formulations. Use of ATX remained stable [20].

A separate study focusing on Sweden, Norway, and Denmark confirmed higher prevalence rates among males, with MPH being the most used medication overall. The fastest increase in use was observed for both MPH and LDX. Guanfacine use increased substantially in Sweden—by 8952% from 2015 to 2020—reaching 4.4 per 1000. Although in Denmark and Norway guanfacine use remained lower, increases of 310% (0.45 per 1000) and 798% (0.67 per 1000) were noted between 2015 and 2020 [21].

#### 3.2.2. Norway

Between 2006 and 2022, among children and adolescents aged 6–17 years, the overall use of ADHD medication increased from 15.9 to 30.3 per 1000. The highest prevalence in both boys and girls was observed in the 13–17 age group, with boys in this group showing the highest rate overall. Notably, in 2022, the prevalence among girls aged 13–17 has approached the levels observed in boys aged 6–12, a group that has consistently held the second-highest position since 2006 [18]. Between 2004 and 2014, a slightly different pattern of ADHD medication use was observed between girls and boys across the life course. Among boys, consumption began to rise around age 6, peaked at age 13, and then declined sharply by age 17. In girls, following an increase in use during preadolescence, a similar sharp decline was not observed. Instead, the values tended to level out over time girls [22].

In adult populations (18–64 years), prevalence increased from 2.4 to 16.9 per 1000, with female use rising almost 10-fold (from 1.9 to 17.6 per 1000) and exceeding male use in the 18–24 group by 2022 [18].

A temporary plateau in prescribing was observed between 2010 and 2020, followed by a post-2020 acceleration [18].

The studies consistently report that prevalence values for females are increasing faster than for males in Norway [18,23,43]. The overall M/F ratio declined from 2.4 to 1.1 between 2006 and 2022 [18]. The increase is particularly pronounced among females aged 18–24 and 25–44, where prevalence rose 8-fold and 18-fold, respectively [43].

MPH remains the most commonly used medication [44]. Following the introduction of ER formulations in 2002, an increase in its use was observed, accompanied by a decline in the IR forms [23]. The second most used medication in 2020 is LDX [44].

#### 3.2.3. Sweden

Before 2016, the highest values were reported in the 10–14 age group. Since 2016, the highest prevalence has been observed in the 15–19 age group, reaching 82.54 per 1000 inhabitants in 2024. However, when analyzed separately by gender, the highest prevalence in 2024 among boys was in the 10–14 age group (91.14 per 1000), whereas among girls it was in the 15–19 age group (83.34 per 1000). Moreover, in 2024, higher prevalence rates were recorded among females compared to males in the 15–19 group and across all adult age groups.

Children aged 0–4 are the only age group that has not shown a consistent increase in prevalence between 2006 and 2024. In both genders, a few years of increase were followed by a period of stabilization and then a decline.

Prevalence values were rising more rapidly among adults and among females. The M/F ratio has gradually decreased from 2.62 in 2006 to 1.07 in 2024.

In the Swedish population, MPH remained the most commonly used substance in ADHD treatment, with its prevalence increasing from 1.41 in 2006 to 12.81 in 2024.

Since the introduction of LDX in 2014, its use has steadily grown, approaching the prevalence of MPH and reaching 11.83 per 1000 in 2024. Notably, when considering LDX use by gender, its prevalence among females surpassed that of MPH in 2024.

It is also worth noting that since 2015, the use of GUA has been steadily increasing, making it the third most commonly prescribed ADHD medication in Sweden after 2022—particularly among boys [42].

#### 3.2.4. Iceland

In 2024, the overall prevalence of ADHD medication use in Iceland reached similar levels for females (62.80 per 1000) and males (62.54 per 1000). The highest prevalence was observed among boys aged 6–17 years, reaching 154.5 per 1000. In the child and adolescent population, the M/F ratio gradually decreased over the years, dropping from 3.11 in 2005 to 1.51 in 2024. Noteworthy, among adults and within all adult age subgroups, prevalence in 2024 was higher among females. The adult M/F ratio began to fall below 1 after 2017.

Across all age groups, MPH was the most frequently used ADHD medication, followed by LDX. After many years of continuous growth, MPH use declined between 2023 and 2024 across the entire population, including both the youth and adult subgroups [46].

#### 3.2.5. Denmark

Between 1999 and 2024, a general increase in prevalence was observed, with the exception of a temporary decline between 2012 and 2016, after which the upward trajectory resumed.

In 2024, the highest overall prevalence was recorded in individuals aged 17 years, at 61.20 per 1000 population. Among females, peak prevalence also occurred at age 17 (67.05 per 1000), whereas among males, the highest prevalence was observed at age 12 (65.07 per 1000). Examination of temporal trends revealed a progressive shift towards older ages at peak prevalence over the study period. Specifically, during 2020–2024, peak prevalence was consistently observed among 17-year-olds; in 2019, among 16-year-olds; in 2015–2018, among 15-year-olds; in 2014, among 13-year-olds; and in 2012–2013, among 12-year-olds. When stratified by gender, the age of peak prevalence exhibited greater temporal stability: among females, it consistently occurred between 17 and 18 years of age, whereas among males, it remained within the 12–14-year range throughout 2012–2024. According to the Danish registry (MEDSTAT), the overall M/F ratio has been steadily declining, nearly equalizing by 2024. MPH was the most frequently used ADHD medication overall and across all age groups, followed by LDX [45].

According to the Danish registry (MEDSTAT), the overall M/F ratio has been steadily declining, nearly equalizing by 2024. MPH was the most frequently used ADHD medication overall and across all age groups, followed by LDX [45].

### 3.3. Europe

#### 3.3.1. Netherlands

Between 1996 and 2006, prevalence among boys (0–19 years) increased from 4.5 to 31.1 per 1000, and among girls from 0.7 to 8.1 per 1000. During this period, the M/F ratio declined from 6.4 to 3.8 [26]. In boys between 2000 and 2007, the highest prevalence was 44.5 per 1000 in the 10–12 age group, while for girls, it peaked at 8.5 per 1000 in the same age range [24].

A study covering 2005–2012 showed that the most pronounced rise was observed in the 15–19 age group. The highest prevalence in 2012 was recorded in 10–14 age group. The M/F ratio declined from 3.87 to 3.04. Prevalence remained low in children aged 0–4 years and decreased further from 1.8 to 0.8 per 1000 over the study period [17].

More recent findings indicate that prevalence among children and adolescents has been declining since 2015. Across all years, boys had higher prevalence than girls in each age group up to age 19. For girls, the highest rates were consistently seen in the 13–19 age group, whereas in boys, prevalence peaked at age 7–12 in 2010 but shifted to 13–19 in later years.

MPH remained the most prescribed substance, with ATX in second position until 2019, after which LDX became more commonly used [25].

#### 3.3.2. Germany

A study based solely on MPH prescriptions found that in 2005–2006, male prevalence was approximately four times higher than female. Peak usage occurred at age 11 for boys and age 10 for girls [27].

According to a nationwide analysis of stimulant prescriptions, overall stimulant prevalence increased from 10.5 per 1000 in 2004 to 19.4 per 1000 in 2010 and remained stable through 2012 [28].

An international comparative study covering the period 2005–2012 stated that the highest ADHD medication prevalence in Germany occurred in the 10–14 age group in 2012. The largest relative increase was observed among adolescents aged 15–19, where prevalence rose nearly 6-fold. In contrast, a significant decrease was noted among children aged 0–4. The M/F ratio declined from 4.51 to 3.60. MPH accounted for the majority of prescriptions (90.3–91.0%), while ATX held a smaller share (8.6–9.6%) by 2012 [17].

#### 3.3.3. France

Between 2005 and 2011, the number of MPH users increased by 135% among children and 343% among adults. The highest proportion of users remained in the 6–12 age group. In contrast, the largest relative increase occurred among adults aged 35–49, rising from 5.9% to 12.3% over the same period. A gradual increase in the proportion of girls among child users was also observed, from 15.1% in 2005 to 20.7% in 2011 [29].

Prevalence of ADHD medication use in children and adolescents (3–18 years) increased from 1.9 per 1000 in 2006 to 3.9 per 1000 in 2014. Among adults, prevalence rose from 0.1 to 0.2 per 1000 in the same period [16].

Among individuals aged 0–25 years, the prevalence increased from 3.1 per 1000 in 2016 to 5.6 per 1000 in 2022. The highest rates were consistently observed in children aged 6–12 years, where prevalence rose from 6.8 to 11.6 per 1000, followed by adolescents aged 13–17 years (from 5.7 to 9.5 per 1000). Use among young adults (18–25 years) also increased more than twofold (from 1.0 to 2.7 per 1000). Across the study period, both absolute and relative increases were observed in males and females, though the rise was more pronounced in females (RR, 15%; 95% CI, 13–18%) compared with males (RR, 9%; 95% CI, 6–12%). Despite this faster relative growth, methylphenidate prescriptions remained substantially more common among males, who accounted for approximately three-quarters of users [30].

#### 3.3.4. Slovenia

Among children and adolescents between 2003 and 2015 MPH had higher overall prevalence than ATX throughout the study period. The prevalence of MPH increased overall and was the highest in 6–12 age group. A 2-fold increase was noted specifically in the 13–17 age group. In contrast, ATX showed a relatively greater increase in use than MPH, particularly in adolescents aged 13–17 [32].

A 10-fold increase in MPH and ATX use prevalence was observed among adults aged 18–24 between 2003 and 2015. For ATX, the difference between age groups was less pronounced than for MPH. Additionally, ATX was more commonly used than MPH among adults aged 25–49 and over 50 [31].

#### 3.3.5. Ireland

MPH was the most frequently prescribed ADHD medication, with prevalence rising from 3.68 per 1000 in 2002 to 7.51 per 1000 in 2011. DEX use remained stable over the study period. ATX introduced in 2007, quickly became the second most commonly prescribed ADHD medication.

Boys aged 5–11 and 12–15 were significantly more likely to receive psychostimulants than girls in the same age groups throughout the study period. Among girls, the prevalence was initially similar between these age categories, but from 2009 onward, higher rates were observed in the 12–15 age group compared to the 5–11 age group [33].

#### 3.3.6. United Kingdom (UK)

From 2003 to 2008, annual prevalence increased among children, adolescents, and adults over 45 years of age, with the largest increases observed in the 18–24 and 25–45 age groups. Prevalence was lower in females across age groups, but growth was faster for females in groups: 6–12, 13–17, and 25–45. The opposite pattern was seen in 18–24 and ≥45 [35].

Between 2005 and 2012, among children and adolescents, the prevalence of ADHD medication use increased most rapidly in the 15–19 age group, while remaining highest among those aged 10–14 years. A decline in the M/F ratio was observed; however, in 2012, it still reached a relatively high value of 5.00 [17].

Between 2000 and 2018, the highest prevalence was recorded in 10–15 age group. The proportion of individuals prescribed ADHD medication increased markedly in both children (4-fold in boys and 9-fold in girls) and adults (30-fold in males and 15-fold in females), with increases observed in all age groups except 3–5-year-olds, for whom a decrease was recorded [36].

In 2018, the highest prevalence was recorded among boys aged 10–16, at 23.69 per 1000. Among females, the highest prevalence was also observed in the 10–16 age group. That year, prevalence rates were higher in males than in females across all age groups, except for the 3–5 age group. The fastest growth among adults was observed in those aged 18–29. In the 6–9, 10–16, and 16–17 age groups, prevalence increased more rapidly among females, whereas in all adult age groups, the increase was steeper among males [36].

MPH was the dominant ADHD medication among children (0–15), accounting for approximately 94% of all ADHD prescriptions. Prevalence among children rose from 0.15 per 1000 in 1995 to 5.07 per 1000 in 2008, then stabilized at 5.11 per 1000 in 2013. IR-MPH was progressively replaced by ER-MPH [34]. In 2012, MPH remained the most commonly prescribed ADHD medication in the United Kingdom [17].

#### 3.3.7. Israel

Between 2006–2011, boys were more likely than girls to initiate stimulant treatment, and the highest prevalence in 2011 was observed among children aged 9–11 years (60 per 1000) [41].

### 3.4. Australia

According to the Australian Institute of Health and Welfare, between 2004–2005 and 2018–2019, prevalence rose from 2 to 8 per 1000 population, followed by a sharper increase to 22 per 1000 by 2023–2024. The male 12–17 age group exhibited the highest overall rates of ADHD medication use, increasing from 10 per 1000 population in 2004–2005 to 87 per 1000 in 2023–2024. MPH was consistently the most prescribed medication, while LDX emerged as the second most dispensed in recent years [47].

Throughout the period covered in the report, males were more likely than females to receive ADHD medications, but the gender gap narrowed considerably. Between 2018–2019 and 2023–2024, male rates doubled (from 11 to 26 per 1000), while female rates increased 5-fold (from 4 to 19 per 1000). By 2022–2023, female rates surpassed male rates in the 18–24 and 25–44 age groups. In recent years, LDX has become the most frequently prescribed medication among patients in the 18–24 and 25–44 age groups [47].

In earlier years (2007–2015), DEX was the most prevalent ADHD medication among adults [39].

### 3.5. Asia

#### 3.5.1. South Korea

Between 2007 and 2011, males consistently represented at least four times as many cases as females. However, the relative increase in prevalence was greater among females than males. The highest prevalence was observed in the 6–12 age group. While prevalence decreased in children under 6, it increased in older age groups, with the largest relative increase recorded among adolescents aged 13–17 [40].

#### 3.5.2. Japan

Between 2010 and 2015, the prevalence of ADHD medication (MPH and ATX) use increased substantially among both children/adolescents (aged 3–18 years) and adults (>18 years). In the pediatric population, prevalence rose from 2.9 per 1000 in 2010 to a peak of 6.5 per 1000 in 2014, followed by a slight decline to 5.4 per 1000 in 2015. Among adults, the prevalence remained low but showed a steady increase—from 0.03 per 1000 in 2010–2011 to 0.5 per 1000 by 2014–2015 [16].

#### 3.5.3. Taiwan

Between 2002 and 2010, the prevalence of ADHD medication (MPH and ATX) use in Taiwan increased markedly, particularly among children and adolescents aged 3–18 years. In this group, prevalence rose from 3.1 per 1000 in 2002 to 15.4 per 1000 in 2010. Among adults (>18 years), prevalence remained much lower and relatively stable, increasing modestly from 0.2 per 1000 in 2002 to 0.4 per 1000 in 2010.

## 4. Discussion

This narrative review provides a synthesis of trends in ADHD medication use across multiple regions worldwide. The data consistently demonstrate an overall increase in ADHD medication prevalence in most studied countries, with the sole exception of the Netherlands, where recent years have seen a decline. To enhance clarity, the discussion distinguishes between descriptive findings, plausible explanatory hypotheses, and speculative interpretations regarding observed trends.

Descriptive findings consistently demonstrate marked cross-country differences in ADHD medication prevalence. The highest values were reported in the US, the Nordic countries—particularly Iceland—as well as in Canada and the Netherlands. Lower prevalence rates were generally found in some Western European countries, including the UK, France, Slovenia, Ireland, and Spain, as well as in Asian countries and Australia. Given that no differences in ADHD prevalence have been found across different regions of the world [48,49,50] and that ADHD prevalence has remained stable over time [49], the observed cross-national differences and increases in ADHD medication use are likely attributable to other factors.

The highest prevalence of ADHD medication use was observed in older children and adolescents, followed by a gradual decline across successive adult age groups. This is consistent with findings that the prevalence of ADHD symptoms decreases with age in adults [6]. In most countries, the age of peak prevalence of ADHD medication use has shifted upwards over time. For example, Denmark demonstrated a progressive increase in the peak age from early adolescence (12 years) in 2012–2013 to late adolescence (17 years) by 2020–2024. In Sweden, Norway, and the Netherlands, similar upward shifts were observed, whereas in South Korea, the UK, and Canada, the fastest increases were recorded among adolescents and young adults. The growth in adult use is a notable finding, with some of the fastest increases seen in young adults. Declines in prevalence among preschoolers have been reported in Sweden, the Netherlands, Germany and the US, as well as in the UK.

In nearly all included countries, males exhibited higher prevalence rates than females, particularly during childhood and adolescence. However, a recurring pattern across several regions—including the US, Canada, the Nordic countries, Australia, and parts of Western Europe—was a faster relative increase in ADHD medication use among females. Evidence from several studies indicates that the prevalence among females has already surpassed that of males within adult age groups.

To better understand these descriptive patterns, we next consider plausible but not definitively proven explanatory factors that may account for observed differences in ADHD medication use across countries and over time.

Stimulants were the most frequently used pharmacological treatment for ADHD, a trend likely attributed to their well-established efficacy and safety profile [51], as well as to recommendations of national guidelines [1,2,3,4]. The rise in LDX prescribing is potentially associated with its long duration of action, which may improve both adherence and persistence. For example, a retrospective analysis using the US MarketScan claims database reported that LDX was associated with superior treatment adherence compared with other ADHD medications, including both stimulant and non-stimulant agents [52]. Furthermore, NICE guidelines recommend LDX as a second-line option [1].

Since 2014, ADHD in the US has been diagnosed according to the DSM-5 (Diagnostic and statistical manual of mental disorders) criteria, and in 2022 the ICD-11 (International statistical classification of diseases and related health problems) came into effect, replacing the ICD-10 used in most European countries. Both the DSM-5 and the ICD-11 broaden the diagnostic criteria for ADHD by extending the permissible age of symptom onset and reducing the number of required symptomatic criteria, thereby enabling diagnosis in a wider group of patients [53,54,55].

Furthermore, individual countries apply distinct national guidelines and recommendations regarding the timing of pharmacological treatment initiation. For children and adolescents over 5 years of age, the NICE (The National Institute for Health and Clinical Excellence) guidelines recommend initiating pharmacological treatment if non-pharmacological interventions prove ineffective [1]. In contrast, the AAP (The American Academy of Pediatrics) guidelines advise implementing pharmacological treatment in combination with non-pharmacological interventions [2].

Differences in access to mental health services or financial resources, as well as disparities in reimbursement eligibility criteria, may also play a role [56]. In Sweden, a marked increase in guanfacine use has been linked to improved access to mental health services, which facilitates more frequent consultations and, consequently, a higher likelihood of initiating second-line pharmacotherapy [21]. A longitudinal trend study found that the multinational increase in ADHD medication consumption appeared to be largely driven by high-income countries [57]. In the US, comparative analyses using two large national databases—MarketScan (privately insured) and Medicaid (low-income populations)—showed higher ADHD medication use among Medicaid enrollees for both children and adults. These differences are likely shaped by variations in medication affordability, access to behavioral therapies, and population demographics. Families with greater financial resources and educational capital are more likely to access psychosocial interventions and private behavioral therapy, whereas those with limited means may depend more heavily on pharmacological treatment due to constraints within the public healthcare and educational systems [16].

Similar socioeconomic disparities have been documented across Europe and Australia. In England, a recent nationwide analysis at the Integrated Care Board level found a significant positive linear association between ADHD prescribing rates and regional deprivation (estimate = 1.11, SE 0.35, *p* < 0.01), indicating higher prescribing intensity in more deprived areas [58]. In Australia, longitudinal study (2002–2015) showed that children from socioeconomically disadvantaged families had significantly lower medication coverage (β = 4.0; 95% CI 0.2–7.8; *p* = 0.04), reflecting poorer treatment continuity and adherence over time [59].

A Swedish multilevel population-based study in Stockholm County found that neighborhood-level socioeconomic disadvantage was positively associated with ADHD medication utilization among school-aged children, even after controlling for individual-level characteristics [60]. This suggests that structural factors linked to deprivation—such as educational stressors, limited social support, and differences in healthcare-seeking behavior—may influence diagnostic and prescribing practices.

In Denmark, a national register-based cohort study further highlighted strong regional inequalities in ADHD prescribing among socially disadvantaged children. The incidence of ADHD medication prescribing was more than fourfold higher among children of single parents with low educational attainment in the North region compared with the South region, and twice as high as in the Capital region. These differences persisted even after adjusting for demographic and socioeconomic factors, pointing to regional variations in clinical practice and potentially unequal access to non-pharmacological care [61].

Taken together, these findings emphasize that socioeconomic and contextual inequalities shape both access to and patterns of ADHD pharmacotherapy. In many settings, children and adults from deprived or marginalized backgrounds are more likely to receive pharmacological treatment but may experience lower adherence and limited access to complementary behavioral interventions. Conversely, in higher-income populations, lower medication prevalence may partly reflect better access to alternative or preventive care rather than reduced clinical need.

Social attitudes toward pharmacological treatment, as well as legal and regulatory restrictions, are also relevant. For instance, in the Netherlands, the implementation of the Youth Act in 2015—which decentralized child and adolescent mental health services and explicitly aimed to reduce medicalization—was followed by a marked decline in ADHD medication dispensing among youth. The reform sought to “de-medicalize” mental healthcare for children and adolescents by reducing reliance on psychotropic medication for managing behavioral problems and promoting non-pharmacological treatment methods. Recent analyses of the Dutch IADB.nl database showed that between 2015 and 2022, overall ADHD medication dispensing prevalence declined from 45.7 to 35.2 per 1000 youths, mainly due to a reduction in new prescriptions among children aged 7–12 years, while incidence increased modestly among adolescent females. These findings suggest that the post-2015 decline in medication use likely reflects the intended effects of the Youth Act’s de-medicalization agenda, although other concurrent factors—such as the DSM-5 diagnostic revisions, the COVID-19 pandemic, and medication shortages—may also have contributed to the observed changes [25]. Changes in regulatory communications have also influenced ADHD prescribing patterns. For example, following the U.S. Food and Drug Administration’s (FDA) 2005 Public Health Advisory on “Suicidal Thinking in Children and Adolescents Being Treated with Strattera (Atomoxetine)” and the subsequent addition of a boxed warning to atomoxetine’s label, a temporary shift in prescribing trends was observed. An interrupted time-series analysis using the IMS LifeLink Health Plan Claims database (2004–2007) demonstrated a significant short-term decline in new atomoxetine initiations across all age groups, with the most pronounced reduction among adults (−11.9%, 95% CI: 3.1–20.7%) immediately after the warning was issued. However, no sustained long-term decrease was detected, suggesting that initial prescriber caution was followed by stabilization of atomoxetine use once safety concerns were more clearly contextualized [62]. In Denmark, a decrease in prescribing was observed after 2010, when the Danish Medicines Agency issued a report warning against ADHD medication use in adults due to insufficient evidence of safety [21]. In Australia, for instance, an expansion of the PBS listing in February 2021 allowed adults diagnosed with ADHD after the age of 18 to access publicly subsidized lisdexamfetamine for the first time. An interrupted time-series analysis based on PBS dispensing data demonstrated that this policy change was followed by an immediate 37% increase in monthly LDX dispensings, and a continued rise of approximately 5% per month thereafter. The initial surge likely reflected the transition of patients from privately funded to subsidized prescriptions, while the sustained increase suggested growing awareness and diagnosis of adult ADHD, as well as prescriber and patient preference for LDX owing to its convenient once-daily dosing and perceived lower abuse potential [63].

The increase in ADHD medication consumption among adults may also be explained by a shift in the perception of ADHD—from a disorder affecting only the developmental population to a condition that can persist into adulthood [6]. Another relevant factor may be the tendency of individuals diagnosed during childhood or adolescence to continue pharmacological treatment into adulthood. However, a systematic review reported a mean persistence of ADHD pharmacotherapy of only 5.6 months during a one-year follow-up period, which challenges the hypothesis that the rising prevalence in adult age groups is driven by the continuation of medication initiated before the age of 18 [64].

It should also be noted that the COVID-19 pandemic constituted an important factor contributing to the accelerated rise in ADHD medication use observed in recent years [65,66]. This influence may be explained by a worsening of ADHD symptoms due to the social isolation caused by the pandemic [67]. Evidence from interrupted time-series analyses in multiple countries supports this interpretation. In France, an interrupted time-series analysis of national health insurance data including over 20 million individuals aged ≤ 25 years demonstrated a significant post-pandemic increase in methylphenidate prescribing, with relative risks of 1.15 (95% CI, 1.13–1.18) for females and 1.09 (95% CI, 1.06–1.12) for males [30]. This increase coincided with a broader deterioration in youth mental health, reflected by higher rates of psychiatric consultations and hospitalizations for suicide attempts, particularly among adolescent girls.

Similarly, a multinational interrupted time-series study using population-level dispensing data from Norway, Sweden, and Italy found an increased trend in ADHD medication dispensing among adolescents after March 2020 [68]. Following an initial decline during the first lockdown period, dispensing trajectories shifted upward in the post-pandemic phase, particularly in Scandinavian countries, suggesting renewed growth in ADHD treatment demand.

Taken together, these ITS-based findings indicate that the COVID-19 pandemic may have amplified pre-existing upward trends in ADHD medication use—especially among adolescents and young adults—through multiple mechanisms, including disruption of daily structure, reduced access to behavioral therapies, and the worsening of underlying mental health conditions.

In females, ADHD is more frequently diagnosed as the inattentive subtype [69], which, together with the lower severity of disruptive symptoms in girls compared to boys, accounts for the later age at diagnosis [70,71]. Increased awareness among clinicians of the gender-specific clinical presentation of ADHD, together with changes in ICD diagnostic criteria allowing for diagnosis in the absence of clinically significant hyperactivity and impulsivity, has contributed to an increase in ADHD medication use among females and, indirectly, to the overall rise in prevalence, particularly among adolescents and adults.

Among preschoolers, declining prevalence rates in several countries appear consistent with the AAP guidelines from 2011 and 2019 [2,72], and the NICE guidelines from 2018 [53], which recommend prioritizing non-pharmacological interventions and exercising caution when initiating pharmacological treatment in this age group. These recommendations are supported by evidence indicating poorer tolerability of pharmacological therapy in preschool-aged children [73].

Variations in the prescribing frequency of specific ADHD medications may also be driven by differences in marketing authorizations and other local factors. For instance, in Slovenia, atomoxetine (ATX) was the most commonly prescribed ADHD medication in adults, coinciding with the fact that it was the only drug approved for ADHD treatment in the adult population [31].

The increasing use of ADHD medications has also raised concerns regarding the potential risk of psychostimulant misuse and the overdiagnosis of ADHD [74]. However, alongside individuals using stimulants without a formal ADHD diagnosis, there was also a group of patients with a confirmed diagnosis who might have benefited from medication but did not receive it in the US [75].

A large cross-sectional analysis of more than 230,000 secondary school students conducted between 2005 and 2020 demonstrated that the past-year prevalence of nonmedical use of prescription stimulants varied widely across schools, ranging from 0% to over 25%. Importantly, students attending schools with the highest rates of medical stimulant use had approximately 36% greater odds of engaging in nonmedical use compared with schools where no stimulant therapy was reported [76]. These findings suggest that greater population-level exposure to stimulant medications may increase opportunities for diversion and nonmedical use among peers.

Among adults, similar patterns have been observed. A recent national survey covering 2019–2022 found that approximately one in four adults using prescription stimulants reported misuse, and nearly 9% met criteria for prescription stimulant use disorder. The risk of misuse was notably higher among individuals treated with amphetamine-based medications compared with methylphenidate, and the highest misuse rates were observed among young adults aged 18–25 years. Although prescribing increased most rapidly among middle-aged women, this group showed comparatively lower misuse rates [77].

Together, these findings indicate that the rise in ADHD pharmacotherapy, while reflecting improved access to treatment, may also be accompanied by an expansion of misuse and diversion—particularly among younger populations and in settings where stimulant availability is high. This underscores the importance of balancing accessibility with appropriate monitoring, patient education, and prescriber training to mitigate the risks of nonmedical use and stimulant use disorder.

Beyond data-driven and policy-related explanations, several more speculative factors may also contribute to the observed cross-national differences in ADHD medication use.

Cultural attitudes toward mental health and pharmacological treatment likely play an important role. American and Scandinavian societies demonstrated greater acceptance of psychopharmacological treatment [78,79] compared with certain European countries, such as Italy [80] and France [81], as well as Asian societies [82].

In Western contexts, pharmacological treatment is sometimes perceived not only as a means of managing illness but also as a tool for improving everyday functioning and performance. For instance, students use stimulants to improve their academic outcomes [83].

Western societies have been observed to view pharmacological interventions as a means of addressing everyday life challenges [84], which may contribute to the increased use of psychotropic medications.

The influence of the pharmaceutical industry also played a role in shaping these global patterns. Advances in drug technologies led to the development of formulations that increasingly met patients’ needs, such as the introduction of ER stimulant preparations, which progressively replaced IR formulations [20,34]. The marketing strategies of pharmaceutical industry, aimed at maximizing profit, contributed to a social narrative that expanded the boundaries of what was defined as illness and promoted the use of medications beyond the scope of strictly medical necessity [85].

## 5. Strengths and Limitations

Nonetheless, several limitations of our review should be acknowledged. The included studies employed distinct methodologies. Age groups were defined using different ranges, and medications were analyzed either collectively or stratified by individual active compounds. Furthermore, analyses were based on diverse data sources, including nationwide registers, health insurance provider data, population-based surveys, commercial databases, and governmental records. In some publications, data on ADHD medication use were not linked to information on recorded ADHD diagnoses. What’s more, there was a paucity of literature describing ADHD medication utilization trends in recent years. These methodological and contextual differences limit the ability to generalize findings unequivocally to all countries, hinder direct cross-national comparisons, and impede the identification of factors influencing the development of trends.

To enhance transparency and allow readers to assess the robustness of the findings, we performed a descriptive appraisal of the quality of included data sources, as described in methods section. Importantly, the overall temporal trends and cross-country patterns remained consistent when considering only high-quality national registries, suggesting that our core conclusions are robust despite heterogeneity in data source quality.

Although prevalence is an informative population-level indicator, it offers limited value for drawing conclusions about clinical practice or medication safety without considering adherence and persistence. Only about 23% of children and adolescents achieve good adherence at 12 months, with an average persistence of roughly 170 days, suggesting that discontinuation and inconsistent use are common across settings. These findings, derived from a recent systematic review, indicate that increasing prevalence of ADHD medication use may not necessarily translate into improved clinical outcomes or sustained therapeutic engagement [64].

While this review identifies consistent global patterns in ADHD medication use, important evidence gaps remain. Most available data are descriptive and lack causal inference, limiting the ability to determine whether observed trends reflect policy impacts, true increases in treatment need, or shifts in diagnostic and prescribing practices. Future studies should employ longitudinal and quasi-experimental designs to clarify these relationships and inform evidence-based policy.

The adoption of a narrative review format facilitated the synthesis and comparative analysis of studies employing disparate methodologies and drawing upon varied data sources, thereby allowing for a more comprehensive appraisal of global trends in ADHD medication use.

## 6. Policy and Research Recommendations

The observed global variation in ADHD medication use underscores the need for coordinated policy and research responses. From a policy perspective, strengthening diagnostic competencies within primary care and improving access to evidence-based non-pharmacological therapies—particularly behavioral and psychoeducational interventions—should remain priorities. Establishing active pharmacovigilance and prescription-monitoring systems could help mitigate diversion and nonmedical stimulant use. Furthermore, harmonizing age- and sex-stratified surveillance indicators across countries would enable more accurate cross-national comparisons and inform equitable resource allocation.

From a research perspective, future studies should aim to harmonize data collection and address current evidence gaps. Longitudinal cohort studies linking diagnostic records with prescription registries would help determine whether increasing adult medication use reflects new-onset adult ADHD or continuation from childhood treatment. Analyses using DDD or prescribing intensity could better capture shifts in medication burden even when prevalence plateaus. Finally, cross-national comparative interrupted time-series designs focusing on guideline revisions, reimbursement policy changes, or legislative reforms could strengthen causal inference regarding drivers of medication trends. Research into the social determinants of ADHD treatment—including socioeconomic status, educational background, and rural–urban disparities—remains essential to ensure that increased access translates into equitable and appropriate care.

## Supplementary Materials

The following supporting information can be downloaded at: https://www.mdpi.com/article/10.3390/jcm14207338/s1, S1: Search string; Table S1: Characteristics and Quality Assessment of Studies Reporting the Prevalence of ADHD Medication Use.

## Abbreviations

The following abbreviations are used in this manuscript:

AAP	The American Academy of Pediatrics
AAPC	Average annual percentage change
AIHW	The Australian Institute of Health and Welfare
APC	Annual percentage change
ADHD	Attention-deficit/hyperactivity disorder
AMP	Amphetamine
ATX	Atomoxetine
CLO	Clonidine
DDD	Defined daily dose
DEX	Dexamfetamine
DEX-MPH	Dexmethylphenidate
DSM	Diagnostic and statistical manual of mental disorders
ER	Extended release
GUA	Guanfacine
ICD	International statistical classification of diseases and related health problems
IR	Immediate release
LDX	Lisdexamfetamine
M/F ratio	Male/Female ratio
MPH	Methylphenidate
NICE	The National Institute for Health and Clinical Excellence
UK	United Kingdom
US	United States
