# Supplementary material for: Global Trends in ADHD Medication Use: Multiple Contexts and Rising Concerns—A Narrative Review
Source: J Clin Med. 2025 Oct 17;14(20):7338. doi: 10.3390/jcm14207338 (PMC12565614; doi:10.3390/jcm14207338)
Supplement: Supplementary file 1 [file jcm-14-07338-s001.zip › jcm-3899189-supplementary.pdf]

Supplementary table S1. Overview of included studies with details on the operationalization of ADHD medication use prevalence and data quality assessment

Study	Years covered	Data source	Type of source	Numerator (counted for prevalence of ADHD medication use)	Denominator (counted for prevalence of ADHD medication use)	Author-applied adjustments	Data quality
Lopez-Leon, Sandra et al. Psychotropic medication in children and adolescents in the United States in the year 2004 vs 2014. [1]	2004 and 2014	MarketScan Commercial Claims and Medicare database and the MarketScan Medicaid database	Administrative claims database (commercial and public)	the number of children with ≥1 prescription	the total number of children in the database	Conversion to rates per 1000	Moderate
Zuvekas SH, Vitiello B. Stimulant medication use in children: a 12-year perspective. [2]	1996-2008	The Medical Expenditure Panel Survey (MEPS)	a survey	the number of children <19 with ≥1 stimulant prescription	all children <19 in MEPS sample, weighted to represent the US civilian non-institutionalized population	Conversion to rates per 1000	Low
Zuvekas SH, Vitiello B, Norquist GS. Recent trends in stimulant medication use among U.S. children. [3]	1997–2002	The Medical Expenditure Panel Survey (MEPS)	a survey	the number of children <19 with ≥1 stimulant prescription	all children <19 in MEPS sample, weighted to represent the US civilian non-institutionalized population	Conversion to rates per 1000	Low
Moore TJ, Wirtz PW, Kruszewski SP, Alexander GC. Changes in medical use of central nervous system stimulants among US adults, 2013 and 2018: a cross-sectional study. [4]	2013 and 2018	The Medical Expenditure Panel Survey (MEPS)	a survey	the number of adults ≥19 with ≥1 stimulant prescription	all adults ≥19 in MEPS sample, weighted to represent the US civilian non-institutionalized population	Conversion to rates per 1000	Low

Cox ER, Halloran DR, Homan SM, Welliver S, Mager DE. Trends in the prevalence of chronic medication use in children: 2002-2005. [5]	2002 and 2005	Express Scripts, Inc.	Administrative claims database (commercial)	the number of children (5–19) with ≥1 prescription	average number of enrolled children in the same quarter	none	Moderate
Danielson ML, Bohm MK, Newsome K, et al. Trends in Stimulant Prescription Fills Among Commercially Insured Children and Adults — United States, 2016–2021. MMWR Morb Mortal Wkly Rep 2023;72:327–332. [6]	2016-2021	The Merative MarketScan Commercial Database	Administrative claims database (commercial)	the number of persons with ≥1 prescription stimulant fill during the year	Enrollees aged 5–64 years, continuously enrolled throughout the calendar year.	Conversion to rates per 1000	Moderate
Raman, S. R., Man, K. K. C., Bahmanyar, S., Berard, A., Bilder, S., Boukhris, T., et al. Trends in attention-deficit hyperactivity disorder medication use: a retrospective observational study using population-based databases. [7]	2001 - 2015	Multiple national prescription registries and claims databases, as reported in Raman et al., 2018 [7]	Primary care EHR databases, administrative claims databases, and national prescription registries (multi-country study).	the annual prevalence of each medication was expressed as a percentage, per 100 individuals	As defined in each national dataset (e.g., total population of children/adolescents or adults, depending on the country). For details, see original study table in in Raman et al., 2018 [7].	Conversion to rates per 1000	Moderate

Bachmann CJ, Wijlaars LP, Kalverdijk LJ, Burcu M, Glaeske G, Schuiling-Veninga CCM, Hoffmann F, Aagaard L, Zito JM. Trends in ADHD medication use in children and adolescents in five western countries, 2005-2012. [8]	2005-2012	This multinational study used national or regional administrative and prescription databases	Administrative and prescription databases (Denmark: national prescription registry; Germany: statutory health insurance claims; Netherlands: community pharmacy dispensing; UK: primary care prescribing database; USA: public insurance claims	the number of children and adolescents (0–19 years) with one or more dispensing	Difficult to determine from the available description	Conversion to rates per 1000	Moderate
Hartz I, Madsstuen NHH, Andersen PN, Handal M, Odsbu I. Nationwide trends in the use of ADHD medications in the period 2006-2022: a study from the Norwegian prescription database. [9]	2006-2022	the Norwegian Prescription Database (NorPD)	National prescription registry	the number of individuals with ≥1 dispensed ADHD medication in a calendar year	All residents of Norway aged 6–64 years with a valid personal identification number, counted as of January 1 each year (2006–2022)	none	High
Furu K, Karlstad Ø, Zoega H, Martikainen JE, Bahmanyar S, Kieler H, Pottegård A. Utilization of Stimulants and Atomoxetine for Attention-Deficit/Hyperactivity Disorder among 5.4 Million Children Using Population-Based Longitudinal Data. [10]	2008-2012	Norwegian Prescription Database, Danish National Prescription Registry, Swedish Prescribed Drug Register, Finnish Prescription Register, Icelandic Medicines Registry	National prescription registry	the number of children with ≥1 dispensed ADHD drug in a calendar year	Number of children (0–17 years) in each country at the beginning of each calendar year	none	High

Karlstad Ø, Zoëga H, Furu K, Bahmanyar S, Martikainen JE, Kieler H, Pottegård A. Use of drugs for ADHD among adults-a multinational study among 15.8 million adults in the Nordic countries. [11]	2008-2012	Norwegian Prescription Database, Danish National Prescription Registry, Swedish Prescribed Drug Register, Finnish Prescription Register, Icelandic Medicines Registry	National prescription registry	the number of individuals aged 18–64 with ≥1 dispensed ADHD drug in a calendar year	Gender- and age-specific population aged 18–64 years in each country, retrieved from national population registers (as of each calendar year)	none	High
Sørensen AMS, Wesselhöeft R, Andersen JH, Reutfors J, Cesta CE, Furu K, Hartz I, Rasmussen L. Trends in use of attention deficit hyperactivity disorder medication among children and adolescents in Scandinavia in 2010-2020. [12]	2010-2020	Norwegian Prescription Database, Danish National Prescription Registry, Swedish Prescribed Drug Register	National prescription registry	the number of individuals aged 5–19 with ≥1 dispensed ADHD medication during the year	The number of individuals aged 5–19 years in each country per year by January 1st	none	High
Hartz I, Skurtveit S, Steffenak AK, Karlstad O, Handal M. Psychotropic drug use among 0-17 year olds during 2004-2014: a nationwide prescription database study. [13]	2004-2014	Norwegian Prescription Database	National prescription registry	the number of individuals <18 years with ≥1 dispensed psychotropic drug during the year	All inhabitants <18 years in Norway as of July 1 each year	none	High
Lillemoen PK, Kjosavik SR, Hunskår S, Ruths S. Prescriptions for ADHD medication, 2004-08. [14]	2004-2008	Norwegian Prescription Database	National prescription registry	the number of individuals who collected ≥1 ADHD medication prescription in a year	Total number of inhabitants in Norway	none	High

Hodgkins P, Sasané R, Meijer WM. Pharmacologic treatment of attention-deficit/hyperactivity disorder in children: incidence, prevalence, and treatment patterns in the Netherlands. [15]	2000-2007	PHARMO Record Linkage System	Population-based integrated medical record and pharmacy database	the number of children (6–17 y) with ≥1 dispensed ADHD medication during a given year	total population aged 6–17 that year	none	Moderate
Ringeling, L.T., Srivastava, A., Gangapersad, R.N. *et al.* ADHD medication dispensing trends in Dutch youth before and after the implementation of the Youth Act. [16]	2010-2022	InterAction Database (IADB)	Community pharmacy dispensing database (population-based administrative database)	the number of users	total underlying population present in the IADB database	none	Moderat
Trip AM, Visser ST, Kalverdijk LJ, de Jong-van den Berg LT. Large increase of the use of psycho-stimulants among youth in the Netherlands between 1996 and 2006. [17]	1996-2006	InterAction Database (IADB)	Community pharmacy dispensing database (population-based administrative database)	the number of children/adolescents (0–19 y) with ≥1 psychostimulant prescription in a calendar year	All children and adolescents aged 0–19 years included in the IADB.nl database for each calendar year between 1996 and 2006	none	Moderate
Kraut AA, Langner I, Lindemann C, Banaschewski T, Petermann U, Petermann F, Mikolajczyk RT, Garbe E. Comorbidities in ADHD children treated with methylphenidate: a database study. [18]	2004-2006	Data from four statutory health insurance companies	Administrative claims database (public, multi-payer system)	the number of insured persons aged 3–17 with ≥1 filled methylphenidate prescription during a study year	All insurees aged 3–17 years with valid data on birth year, sex, and residence in Germany, insured at least one day in the respective study year	none	Moderate

Abbas S, Ihle P, Adler JB, Engel S, Günster C, Linder R, Lehmkuhl G, Schübert I. Psychopharmacological Prescriptions in Children and Adolescents in Germany. [19]	2004–2012	Data from two statutory health insurance companies	Administrative claims database (public, multi-payer system)	the number of continuously insured children and adolescents with at least one prescription for a psychotropic medication within a given year	continuously insured children and adolescents in the relevant year	Conversion to rates per 1000	Moderate
Ehrhardt C, Boucherie Q, Pauly V, Braunstein D, Ronflé E, Thirion X, Frauger E, Micallef J. Methylphenidate: Gender trends in adult and pediatric populations over a 7year period. [20]	2005-2011	French general health insurance system (FGHIS)	Administrative claims database (public, multi-payer system)	the number of patients with at least one methylphenidate dispensing during the calendar year	All individuals covered by the French General Health Insurance System (FGHIS) in the two included regions	none	Moderate
Fond G, Pauly V, Brousse Y, et al. Mental Health Care Utilization and Prescription Rates Among Children, Adolescents, and Young Adults in France. [21]	2016-2023	French National Health Insurance Database (SNDS)	National health insurance claims database	individuals aged 0–25 years with ≥1 dispensing of a psychotropic medication per year	total population aged 0–25 years in France	none	Moderate
Stuhec, M., Locatelli, I. Attention deficit hyperactivity disorder pharmacotherapy in Slovenian adults: a population-based study. [22]	2003–2015	Health Insurance Institute of Slovenia (NHI) database	Administrative claims database (public, single -payer system)	the number of adults with at least one prescription for ADHD medication in a given year	Adult population (≥18 years) of Slovenia by age group	Conversion to rates per 1000	Moderate
Stuhec M, Locatelli I. Age-related pharmacotherapy of attention deficit hyperactivity disorder in Slovenia in children and	2003-2015	Health Insurance Institute of Slovenia (NHI) database	Administrative claims database (public, single -payer system)	the number of children and adolescents (<18 years) who were prescribed at least one ADHD drug in a given calendar year	the total number of children and adolescents in the population for that year	none	Moderate

adolescents: A population-based study. [23]							
Boland F, Galvin R, Reulbach U, Motterlini N, Kelly D, Bennett K, Fahey T. Psychostimulant prescribing trends in a paediatric population in Ireland: a national cohort study. [24]	2002-2011	GMS (General Medical Services) pharmacy claims database (income-based)	Administrative claims database (public, multi -payer system)	the number of children with ≥1 dispensed psychostimulant prescription per year	Children aged 0–15 years covered by the GMS scheme	none	Moderate
Beau-Lejdstrom R, Douglas I, Evans SJ, Smeeth L. Latest trends in ADHD drug prescribing patterns in children in the UK: prevalence, incidence and persistence. [25]	1992-2013	CPRD (Clinical Practice Research Datalink)	Electronic medical records – GP prescribing data	the number of children aged under 16 years who received at least one prescription for an ADHD drug during a given calendar year	the mid-year number of children under 16 registered in the CPRD that year, per 1,000	none	Moderate
McCarthy S, Wilton L, Murray ML, Hodgkins P, Asherson P, Wong IC. The epidemiology of pharmacologically treated attention deficit hyperactivity disorder (ADHD) in children, adolescents and adults in UK primary care. [26]	2003-2008	The Health Improvement Network (THIN)	Electronic medical records — GP prescribing data	the number of patients with both a diagnosis of ADHD and at least one prescription for ADH medication within a given calendar yea	the total mid-year source population in that year, per 1,000 individuals	none	Moderate
McKechnie DGJ, O'Nions E, Dunsmuir S, Petersen I. Attention-deficit hyperactivity disorder diagnoses and prescriptions in UK primary care, 2000-	2000-2018	IQVIA Medical Research Data (incorporating data from THIN)	Electronic medical records — GP prescribing data	the number of individuals with ≥2 prescriptions for ADHD medication within one year	the total number of individuals in the eligible cohort	Conversion to rates per 1000	Moderate

2018: population-based cohort study. [27]							
Butt DA, Stephenson E, Kalia S, Moineddin R, Tu K. Patient visits and prescriptions for attention-deficit/hyperactivity disorder from 2017-2021: Impacts of COVID-19 pandemic in primary care. [28]	2017-2021	UTOPIAN Data Safe Haven	Primary care electronic medical record	the number of patients with ≥1 prescription for ADHD medication during the calendar year	All eligible patients aged 5–55 years registered in UTOPIAN practices in a given calendar year who met inclusion criteria	none	Moderate
Morkem R, Patten S, Queenan J, Barber D. Recent Trends in the Prescribing of ADHD Medications in Canadian Primary Care. [29]	2005-2015	Canadian Primary Care Sentinel Surveillance Network (CPCSSN)	Primary care electronic medical record	the number of patients prescribed with ≥1 prescription for ADHD medication during the calendar year	the total number of patients with ≥1 primary care encounter in the same year, per 100	Conversion to rates per 1000	Moderate
Brett J, Karanges EA, Daniels B, et al. Psychotropic medication use in Australia, 2007 to 2015: Changes in annual incidence, prevalence and treatment exposure. [30]	2006-2015	Pharmaceutical Benefits Scheme (PBS)	Administrative claims database of subsidized outpatient prescriptions dispensed at community pharmacies	the number of individuals with ≥1 dispensing of ADHD medication in a calendar year	Adults aged ≥18 years who were long-term concessional beneficiaries continuously eligible for PBS-subsidized medicines	none	High
Song I, Lee MS, Lee E-K, Shin J-Y. Patient and provider characteristics related with prescribing of ADHD medication: Nationwide health insurance claims database study in Korea. [31]	2007-2011	Korea Health Insurance Review and Assessment Service (HIRA)	National administrative claims database (universal, single-payer system)	the number of patients <18 years with ≥1 prescription in a given year	All individuals aged <18 years in Korea	none	High

Hoshen MB, Benis A, Keyes KM, Zoëga H. Stimulant use for ADHD and relative age in class among children in Israel. [32]	2006-2011	Clalit Health Services database	Administrative health insurance claims database (large nonprofit insurer, part of Israel's universal multi-payer system	the number of children who filled ≥1 prescription in a given calendar year	All children aged 6–17 years with at least one full year of continuous Clalit insurance coverage	Conversion to rates per 1000	Moderate
The Swedish Prescribed Drug Registry. [33]	2006-2024	-	National prescription registry	the number of patients with ≥1 dispensed ADHD drug in a calendar year	The number of individuals in each country per year by January 1st	none	High
Norhealth (www.norgeshelsa.no, accessed on 14 July 2025) [34], The Norwegian Prescription Database [35]	2004-2021	-	National prescription registry	the number of patients with ≥1 dispensed ADHD drug in a calendar year	The number of individuals in each country per year by January 1st	none	High
The Danish Health Data Authority, medstat.dk and date. [36]	1999-2024	-	National prescription registry	the number of patients with ≥1 dispensed ADHD drug in a calendar year	The number of individuals in each country per year by January 1st	none	High
The Icelandic Pharmaceutical Database [37]	2005-2024	-	National prescription registry	the number of patients with ≥1 dispensed ADHD drug in a calendar year	The number of individuals in each country per year by January 1st	none	High
The Australian Institute of Health and Welfare (AIHW) (2025) ADHD medications dispensed 2004–05 to 2023–24 [38]	2004-2024	Pharmaceutical Benefits Scheme	Administrative claims database of subsidized outpatient prescriptions dispensed at community pharmacies	the number of individuals with ≥1 dispensing of ADHD medication in a calendar year	All who were long-term concessional beneficiaries continuously eligible for PBS-subsidized medicines	none	High

References

1. Lopez-Leon, S.; Lopez-Gomez, M.I.; Warner, B.; Ruiter-Lopez, L. Psychotropic medication in children and adolescents in the United States in the year 2004 vs 2014. *Daru* **2018**, *26*, 5-10, doi:10.1007/s40199-018-0204-6.
2. Zuvekas, S.H.; Vitiello, B. Stimulant medication use in children: a 12-year perspective. *Am J Psychiatry* **2012**, *169*, 160-166, doi:10.1176/appi.ajp.2011.11030387.
3. Zuvekas, S.H.; Vitiello, B.; Norquist, G.S. Recent trends in stimulant medication use among U.S. children. *Am J Psychiatry* **2006**, *163*, 579-585, doi:10.1176/ajp.2006.163.4.579.
4. Moore, T.J.; Wirtz, P.W.; Kruszewski, S.P.; Alexander, G.C. Changes in medical use of central nervous system stimulants among US adults, 2013 and 2018: a cross-sectional study. *BMJ Open* **2021**, *11*, e048528, doi:10.1136/bmjopen-2020-048528.
5. Cox, E.R.; Halloran, D.R.; Homan, S.M.; Welliver, S.; Mager, D.E. Trends in the prevalence of chronic medication use in children: 2002-2005. *Pediatrics* **2008**, *122*, e1053-1061, doi:10.1542/peds.2008-0214.
6. Danielson, M.L.; Bohm, M.K.; Newsome, K.; Claussen, A.H.; Kaminski, J.W.; Grosse, S.D.; Siwakoti, L.; Arifkhanova, A.; Bitsko, R.H.; Robinson, L.R. Trends in Stimulant Prescription Fills Among Commercially Insured Children and Adults - United States, 2016-2021. *MMWR Morb Mortal Wkly Rep* **2023**, *72*, 327-332, doi:10.15585/mmwr.mm7213a1.
7. Raman, S.R.; Man, K.K.C.; Bahmanyar, S.; Berard, A.; Bilder, S.; Boukhris, T.; Bushnell, G.; Crystal, S.; Furu, K.; KaoYang, Y.H.; et al. Trends in attention-deficit hyperactivity disorder medication use: a retrospective observational study using population-based databases. *Lancet Psychiatry* **2018**, *5*, 824-835, doi:10.1016/S2215-0366(18)30293-1.
8. Bachmann, C.J.; Wijlaars, L.P.; Kalverdijk, L.J.; Burcu, M.; Glaeske, G.; Schuiling-Veninga, C.C.M.; Hoffmann, F.; Aagaard, L.; Zito, J.M. Trends in ADHD medication use in children and adolescents in five western countries, 2005-2012. *Eur Neuropsychopharmacol* **2017**, *27*, 484-493, doi:10.1016/j.euroneuro.2017.03.002.
9. Hartz, I.; Madsstuen, N.H.H.; Andersen, P.N.; Handal, M.; Odsbu, I. Nationwide trends in the use of ADHD medications in the period 2006-2022: a study from the Norwegian prescription database. *BMC Psychiatry* **2024**, *24*, 767, doi:10.1186/s12888-024-06199-9.
10. Furu, K.; Karlstad, O.; Zoega, H.; Martikainen, J.E.; Bahmanyar, S.; Kieler, H.; Pottegard, A. Utilization of Stimulants and Atomoxetine for Attention-Deficit/Hyperactivity Disorder among 5.4 Million Children Using Population-Based Longitudinal Data. *Basic Clin Pharmacol Toxicol* **2017**, *120*, 373-379, doi:10.1111/bcpt.12724.
11. Karlstad, O.; Zoega, H.; Furu, K.; Bahmanyar, S.; Martikainen, J.E.; Kieler, H.; Pottegard, A. Use of drugs for ADHD among adults-a multinational study among 15.8 million adults in the Nordic countries. *Eur J Clin Pharmacol* **2016**, *72*, 1507-1514, doi:10.1007/s00228-016-2125-y.
12. Sorensen, A.M.S.; Wesselhoeft, R.; Andersen, J.H.; Reutfors, J.; Cesta, C.E.; Furu, K.; Hartz, I.; Rasmussen, L. Trends in use of attention deficit hyperactivity disorder medication among children and adolescents in Scandinavia in 2010-2020. *Eur Child Adolesc Psychiatry* **2023**, *32*, 2049-2056, doi:10.1007/s00787-022-02034-2.
13. Hartz, I.; Skurtveit, S.; Steffenak, A.K.; Karlstad, O.; Handal, M. Psychotropic drug use among 0-17 year olds during 2004-2014: a nationwide prescription database study. *BMC Psychiatry* **2016**, *16*, 12, doi:10.1186/s12888-016-0716-x.
14. Lillemoen, P.K.; Kjosavik, S.R.; Hunskar, S.; Ruths, S. Prescriptions for ADHD medication, 2004-08. *Tidsskr Nor Laegeforen* **2012**, *132*, 1856-1860, doi:10.4045/tidsskr.11.1270.
15. Hodgkins, P.; Sasane, R.; Meijer, W.M. Pharmacologic treatment of attention-deficit/hyperactivity disorder in children: incidence, prevalence, and treatment patterns in the Netherlands. *Clin Ther* **2011**, *33*, 188-203, doi:10.1016/j.clinthera.2011.03.001.

16. Ringeling, L.T.; Srivastava, A.; Gangapersad, R.N.; Bos, J.H.J.; de Winter, B.C.M.; Hak, E.; Hillegers, M.H.J.; Koch, B.C.P.; Schuiling-Veninga, C.C.M.; Dierckx, B. ADHD medication dispensing trends in Dutch youth before and after the implementation of the Youth Act (2010-2022). *Eur Child Adolesc Psychiatry* **2025**, doi:10.1007/s00787-025-02791-w.
17. Trip, A.M.; Visser, S.T.; Kalverdijk, L.J.; de Jong-van den Berg, L.T. Large increase of the use of psycho-stimulants among youth in the Netherlands between 1996 and 2006. *Br J Clin Pharmacol* **2009**, *67*, 466-468, doi:10.1111/j.1365-2125.2009.03373.x.
18. Kraut, A.A.; Langner, I.; Lindemann, C.; Banaschewski, T.; Petermann, U.; Petermann, F.; Mikolajczyk, R.T.; Garbe, E. Comorbidities in ADHD children treated with methylphenidate: a database study. *BMC Psychiatry* **2013**, *13*, 11, doi:10.1186/1471-244X-13-11.
19. Abbas, S.; Ihle, P.; Adler, J.B.; Engel, S.; Gunster, C.; Linder, R.; Lehmkuhl, G.; Schubert, I. Psychopharmacological Prescriptions in Children and Adolescents in Germany. *Dtsch Arztebl Int* **2016**, *113*, 396-403, doi:10.3238/arztebl.2016.0396.
20. Ehrhardt, C.; Boucherie, Q.; Pauly, V.; Braunstein, D.; Ronfle, E.; Thirion, X.; Frauger, E.; Micallef, J. Methylphenidate: Gender trends in adult and pediatric populations over a 7year period. *Therapie* **2017**, *72*, 635-641, doi:10.1016/j.therap.2017.05.005.
21. Fond, G.; Pauly, V.; Brousse, Y.; Llorca, P.M.; Cortese, S.; Rahmati, M.; Correll, C.U.; Gosling, C.J.; Fornaro, M.; Solmi, M.; et al. Mental Health Care Utilization and Prescription Rates Among Children, Adolescents, and Young Adults in France. *JAMA Netw Open* **2025**, *8*, e2452789, doi:10.1001/jamanetworkopen.2024.52789.
22. Stuhec, M.; Locatelli, I. Attention deficit hyperactivity disorder pharmacotherapy in Slovenian adults: a population-based study. *Int J Clin Pharm* **2018**, *40*, 341-344, doi:10.1007/s11096-018-0605-0.
23. Stuhec, M.; Locatelli, I. Age-related pharmacotherapy of attention deficit hyperactivity disorder in Slovenia in children and adolescents: A population-based study. *Eur Psychiatry* **2017**, *42*, 129-133, doi:10.1016/j.eurpsy.2017.01.002.
24. Boland, F.; Galvin, R.; Reulbach, U.; Motterlini, N.; Kelly, D.; Bennett, K.; Fahey, T. Psychostimulant prescribing trends in a paediatric population in Ireland: a national cohort study. *BMC Pediatr* **2015**, *15*, 118, doi:10.1186/s12887-015-0435-3.
25. Beau-Lejdstrom, R.; Douglas, I.; Evans, S.J.; Smeeth, L. Latest trends in ADHD drug prescribing patterns in children in the UK: prevalence, incidence and persistence. *BMJ Open* **2016**, *6*, e010508, doi:10.1136/bmjopen-2015-010508.
26. McCarthy, S.; Wilton, L.; Murray, M.L.; Hodgkins, P.; Asherson, P.; Wong, I.C. The epidemiology of pharmacologically treated attention deficit hyperactivity disorder (ADHD) in children, adolescents and adults in UK primary care. *BMC Pediatr* **2012**, *12*, 78, doi:10.1186/1471-2431-12-78.
27. McKechnie, D.G.J.; O'Nions, E.; Dunsmuir, S.; Petersen, I. Attention-deficit hyperactivity disorder diagnoses and prescriptions in UK primary care, 2000-2018: population-based cohort study. *BJPsych Open* **2023**, *9*, e121, doi:10.1192/bjo.2023.512.
28. Butt, D.A.; Stephenson, E.; Kalia, S.; Moineddin, R.; Tu, K. Patient visits and prescriptions for attention-deficit/hyperactivity disorder from 2017-2021: Impacts of COVID-19 pandemic in primary care. *PLoS One* **2023**, *18*, e0281307, doi:10.1371/journal.pone.0281307.
29. Morkem, R.; Patten, S.; Queenan, J.; Barber, D. Recent Trends in the Prescribing of ADHD Medications in Canadian Primary Care. *J Atten Disord* **2020**, *24*, 301-308, doi:10.1177/1087054717720719.
30. Brett, J.; Karanges, E.A.; Daniels, B.; Buckley, N.A.; Schneider, C.; Nassir, A.; Zoega, H.; McLachlan, A.J.; Pearson, S.A. Psychotropic medication use in Australia, 2007 to 2015: Changes in annual incidence, prevalence and treatment exposure. *Aust N Z J Psychiatry* **2017**, *51*, 990-999, doi:10.1177/0004867417721018.
31. Song, I.; Lee, M.S.; Lee, E.K.; Shin, J.Y. Patient and provider characteristics related with prescribing of ADHD medication: Nationwide health insurance claims database study in Korea. *Asia Pac Psychiatry* **2018**, *10*, doi:10.1111/appy.12289.
32. Hoshen, M.B.; Benis, A.; Keyes, K.M.; Zoega, H. Stimulant use for ADHD and relative age in class among children in Israel. *Pharmacoepidemiol Drug Saf* **2016**, *25*, 652-660, doi:10.1002/pds.3962.

33. National Board of Health and Welfare. Socialstyrelsen. National Prescribed Drug Register. Available online: https://sdb.socialstyrelsen.se/if_lak/val_eng.aspx (accessed on 14 July 2025).
34. The Norwegian Institute of Public Health, Norhealth. Available online: www.norgeshelsa.no (accessed on 14 July 2025).
35. The Norwegian Institute of Public Health. The Norwegian Prescription Database. Available online: https://www.norpd.no (accessed on 14 July 2025).
36. Schmidt, M.; Hallas, J.; Laursen, M.; Friis, S. Data Resource Profile: Danish online drug use statistics (MEDSTAT). *Int J Epidemiol* **2016**, *45*, 1401-1402g, doi:10.1093/ije/dyw116.
37. Health, T.D.o. The Icelandic Pharmaceutical Database. Available online: https://island.is/en/pharmaceutical-statistics/adhd-use-of-prescription-medicines (accessed on 14 July 2025).
38. Welfare, A.I.o.H.a. ADHD medications dispensed 2004–05 to 2023–24. Available online: https://www.aihw.gov.au/mental-health/topic-areas/mental-health-prescriptions/adhd-medications-dispensed-overtime (accessed on 14 July 2025).
